# Ethnic variation in validity of classification of overweight and obesity using self-reported weight and height in American women and men: the Third National Health and Nutrition Examination Survey

**DOI:** 10.1186/1475-2891-4-27

**Published:** 2005-10-06

**Authors:** RF Gillum, Christopher T Sempos

**Affiliations:** 1 Centers for Disease Control and Prevention, 3311 Toledo Road, Room 6323, Hyattsville, Maryland, 20782, USA; 2 National Institutes of Health, 6701 Rockledge Drive, Room 3146, Bethesda, Maryland, 20817, USA

**Keywords:** Overweight, Obesity, Hispanics, Mexican Americans, Body weight, Blacks

## Abstract

**Background:**

Few data have been published on the validity of classification of overweight and obesity based on self-reported weight in representative samples of Hispanic as compared to other American populations despite the wide use of such data.

**Objective:**

To test the null hypothesis that ethnicity is unrelated to bias of mean body mass index (BMI) and to sensitivity of overweight or obesity (BMI >= 25 kg/m^2^) derived from self-reported (SR) versus measured weight and height using measured BMI as the gold standard.

**Design:**

Cross-sectional survey of a large national sample, the Third National Health and Nutrition Examination Survey (NHANES III) conducted in 1988–1994.

**Participants:**

American men and women aged 20 years and over (n = 15,025).

**Measurements:**

SR height, weight, cigarette smoking, health status, and socio-demographic variables from home interview and measured weight and height.

**Results:**

In women and Mexican American (MA) men SR BMI underestimated true prevalence rates of overweight or obesity. For other men, no consistent difference was seen. Sensitivity of SR was similar in non-Hispanic European Americans (EA) and non-Hispanic African Americans (AA) but much lower in MA. Prevalence of obesity (BMI >= 30 kg/m^2^) is consistently underestimated by self-report, the gap being greater for MA than for other women, but similar for MA and other men. The mean difference between self-reported and measured BMI was greater in MA (men -0.37, women -0.76 kg/m^2^) than in non-Hispanic EA (men -0.22, women -0.62 kg/m^2^). In a regression model with the difference between self-reported and measured BMI as the dependent variable, MA ethnicity was a significant (p < 0.01) predictor of the difference in men and in women. The effect of MA ethnicity could not be explained by socio-demographic variables, smoking or health status.

**Conclusion:**

Under-estimation of the prevalence of overweight or obesity based on height and weight self-reported at interview varied significantly among ethnic groups independent of other variables.

## Introduction

Obesity, a prevalent and well-established risk factor for cardiovascular disease and diabetes, has been rising in prevalence over recent decades in the United States [1-7]. In 1999–2000, 64% of U.S. adults were found to be overweight or obese, an absolute increase of 8 percentage points from 1988–1994 [5]. Hence surveillance of obesity prevalence is of great public health interest. Because of the high costs of examination surveys, much of the surveillance of obesity is based on self-reported height and weight data, particularly at the state and local level. A number of studies have reported the validity of self-reported weight and height and BMI computed from them in populations of European extraction in the US and elsewhere [8-19]. A recent report noted lower validity of self-reported hypertension in Mexican Americans than in non-Hispanics [20]. However, data are lacking on the validity of self-reported obesity data in the growing Hispanic American population.

Therefore a study was done to test the null hypotheses that there is no association of ethnicity with the validity of BMI or "overweight or obesity" determined from self-reported height and weight and that the estimated measure of association of ethnicity with validity of self-reported overweight or obesity is not confounded by age, gender, education, marital status, region, smoking, and health status in the American population. Data on a large, multi-ethnic, national sample of adults from the Third National Health and Nutrition Examination Survey (NHANES III) were examined.

## Methods

The Third National Health and Nutrition Examination Survey (NHANES III) was conducted in 1988–1994 on a nationwide multi-stage probability sample of 39,695 persons from the civilian, non-institutionalized population aged 2 months and over of the United States excluding reservation lands of American Indians. Mexican Americans and African Americans were oversampled. Of these, 30,818 (78%) were interviewed and examined. Details of the plan, sampling, operation and response have been published as have procedures used to obtain informed consent and to maintain confidentiality of information obtained [20,21].

Demographic data, years of education completed, medical history including self-assessed health status and behavioral information including smoking history were collected by household interview. Participants chose race and ethnicity categories from a card with categories including Mexican American (MA)[21]. Later in the household interview, interviewers asked, "How tall are you without shoes?" (inches) and "How much do you weigh without clothes or shoes?" (pounds). This occurred before they invited participants to take part in an examination and told them they would be measured and weighed.

Examinations were carried out in a mobile examination center. Technicians measured height to the nearest 0.1 centimeter, weight to the nearest 0.01 kg, as described in detail elsewhere [21-23]. Body mass index was computed (BMI = weight/height^2^, kg/m^2^). Obesity was defined as BMI >= 30.0 kg/m^2^. Overweight or obesity was defined as BMI >= 25.0 kg/m^2 ^[5]. Extensive descriptive data on height, weight, BMI, and obesity prevalence have been published elsewhere and will not be duplicated here [23-26].

### Statistical analysis

Of 33,994 interviewed persons, 31,311 were examined (78% of original sample). After exclusion of 14,281 persons under age 20y, 268 pregnant women, and persons with missing height or weight by self report or examination, 15,025 remained for this analysis. Self-reported height and weight were available for over 90% of persons with measured height and weight data. For stratified analysis, a cut-point of 60+ was used because persons 60+ were oversampled. Weighted descriptive statistics and measures of association were computed using the SAS [27-29]. The number of true positives (TP), false positives (FP), true negatives (TN) and false negatives (FN) were used to calculate sensitivity = (TP/(TP+FN))100, positive predictive value (PPV) = (TP/(TP+FP))100, specificity = (TN/(TN+FP))100, and predictive value negative (PVN) = (TN/(TN+FN))100. The BMI difference (error) was computed by subtracting the self-reported value from the measured value. Multivariate linear regression was used for models with BMI difference as a continuous dependent variable. All models controlled for age in years. All statistical testing and variance estimation were performed using the PROC REGRESS procedure for regression models in the SUDAAN system, which takes the complex cluster design of the survey into account [30,31].

## Results

Table 1 compares prevalence of overweight or obesity (BMI >= 25 kg/m^2^) based on BMI computed from self-reported height and weight with that based on BMI computed from measured height and weight. In women, prevalence is consistently and substantially underestimated by self-report data, most markedly among MA both in absolute and relative terms. This was also true of MA men. For other men, no consistent difference was seen. Table 2 shows estimated prevalence of obesity (BMI >= 30 kg/m^2^) by self-report versus measurement. Prevalence is consistently underestimated by self-report, the gap being greater for MA than for other women, but similar for MA and other men. The gap was greater in women than men but varied little with age.

**Table 1:** Prevalence of overweight or obesity defined as body mass index >= 25 kg/m^2^) calculated from self-reported versus measured height and weight by gender, age, and ethnicity: NHANES III.(weighted)

		Ethnicity		
		EA	AA	MA

Men				
20–59y	Self-report	59.2	58.4	59.4
	Measured	58.7	56.4	63.4
	Difference	0.5	0.2	-4.0
60+y	Self-report	61.0	57.6	60.2
	Measured	67.6	58.7	68.9
	Difference	-6.6	-1.1	-8.7
Women				
20–59y	Self-report	39.8	58.9	49.0
	Measured	43.5	64.0	65.1
	Difference	-3.7	-5.1	-16.1
60+y	Self-report	47.0	64.6	50.7
	Measured	57.9	75.6	72.1
		-10.9	-11.0	-21.4
N		6846	4431	3748

EA, Non-Hispanic European American, AA, Non-Hispanic African American, MA, Mexican American

**Table 2:** Prevalence (percent) of obesity defined as body mass index >= 30 kg/m^2^) calculated from self-reported versus measured height and weight by gender, age, and ethnicity: NHANES III.(weighted)

		Ethnicity		
		EA	AA	MA

Men				
20–59y	Self-report	16.2	18.2	18.5
	Measured	19.4	20.5	20.4
	Difference	-3.2	-2.3	-1.9
60+y	Self-report	15.0	17.1	18.7
	Measured	21.5	22.4	22.2
	Difference	-6.5	-5.3	-3.5
Women				
20–59y	Self-report	17.6	30.0	21.6
	Measured	22.1	36.1	33.3
	Difference	-4.5	-6.1	-11.7
60+y	Self-report	16.4	31.2	20.8
	Measured	24.1	40.0	34.4
	Difference	-7.7	-8.8	-13.6
N		6846	4431	3748

EA, Non-Hispanic European American, AA, Non-Hispanic African American, MA, Mexican American WT VOB123003

Tables 3 and 4 show test characteristics for the classification of overweight or obesity based on BMI calculated from self-reported height and weight by age, gender and ethnicity. Sensitivity was similar in non-Hispanic European Americans (EA) and non-Hispanic African Americans (AA) but much lower in MA. Sensitivity was also lower in persons aged 60 years and over than < 60 years and in women than men. The highest sensitivity was in younger EA men (93%) and the lowest in older MA women (69%) (Table 3). PPV was similar across ethnic groups. It was higher in older persons and women. PPV was highest in older EA women (98%) and lowest in younger AA and MA men (89%).

**Table 3:** Sensitivity and positive predictive value (PPV) of overweight or obesity defined as body mass index >= 25 kg/m^2 ^calculated from self-reported height and weight by gender, age, and ethnicity: NHANES III.(weighted)

		Ethnicity		
		EA	AA	MA

Men				
20–59y	Sensitivity	93	92	83
	PPV	92	89	89
60+y	Sensitivity	87	88	82
	PPV	96	90	94
Women				
20–59y	Sensitivity	88	89	72
	PPV	96	97	95
60+y	Sensitivity	80	83	69
	PPV	98	97	95
N		6846	4431	3748

EA, Non-Hispanic European American, AA, Non-Hispanic African American, MA, Mexican American, PPV, positive predictive value

**Table 4:** Specificity and predictive value negative (PVN) of overweight or obesity defined as body mass index >= 25 kg/m^2^) calculated from self-reported height and weight by gender, age, and ethnicity: NHANES III.(weighted)

		Ethnicity		
		EA	AA	MA

Men				
20–59y	Specificity	89	85	82
	PVN	90	89	74
60+y	Specificity	93	86	89
	PVN	77	84	69
Women				
20–59y	Specificity	97	94	93
	PVN	91	83	64
60+y	Specificity	98	91	91
	PVN	78	63	51
N		6846	4431	3748

EA, Non-Hispanic European American, AA, Non-Hispanic African American, MA, Mexican American

Specificity was highest in EA and similar in AA and MA (Table 4). It was higher in women than men and in older than younger men, but varied little with age in women. The highest specificity was in older EA women (98%) and the lowest in younger MA men (82%). In men, PVN was similar in EA and AA but much lower in MA within age groups. It was higher in younger than older men. In women, PVN was highest in EA, intermediate in AA and lowest in MA. It was higher in younger than older women, and lower than in men among AA and MA, but not EA women. The highest PVN was in younger EA women (91%) and the lowest in older MA women (51%).

The effect of smoking status, a possible effect modifier, on sensitivity of overweight or obesity based on self-reported height and weight was examined (Appendix Table A1, see Additional file 1). No consistent effect was seen in men. In women, sensitivity tended to be slightly higher in younger non-smokers than in smokers, but higher in older smokers than non-smokers, the difference being large only among older Mexican American women (smokers 82%, non-smokers 66%).

The percentages not overweight by self report (BMI < 25 kg/m^2^) with measured BMI >= 25 kg/m^2^, i.e. false negatives for overweight or obesity, among men and women by age, smoking and ethnicity were examined (Appendix Table 2, see Additional file 1). Rates of false negatives by self-report were slightly higher in non-smokers than in smokers, and in MA than in AA or EA. False negative rates were also higher in older than in younger persons and in less educated than more educated persons (not shown).

Tables 5 and 6 show the test characteristics for self-reported data for classifying persons as obese (BMI >= 30 kg/m^2^). The pattern is similar to that for overweight or obesity. A few exceptions are the similar sensitivity and PVN but lower PPV in MA men compared to other men.

**Table 5:** Sensitivity and positive predictive value (PVP) of obesity defined as body mass index >= 30 kg/m^2 ^calculated from self-reported height and weight by gender, age, and ethnicity: NHANES III.(weighted)

		Ethnicity		
		EA	AA	MA

Men				
20–59y	Sensitivity	77	78	75
	PPV	93	88	83
60+y	Sensitivity	65	67	70
	PPV	93	88	83
Women				
20–59y	Sensitivity	76	77	61
	PPV	95	93	94
60+y	Sensitivity	66	73	57
	PPV	98	93	94
N		6846	4431	3748

EA, Non-Hispanic European American, AA, Non-Hispanic African American, MA, Mexican American

**Table 6:** Specificity and predictive value negative (PVN) of obesity defined as body mass index >= 30 kg/m^2^) calculated from self-reported height and weight by gender, age, and ethnicity: NHANES III.(weighted)

		Ethnicity		
		EA	AA	MA

Men				
20–59y	Specificity	99	97	96
	PVN	95	94	94
60+y	Specificity	99	97	96
	PVN	91	91	92
Women				
20–59y	Specificity	99	97	98
	PVN	93	88	83
60+y	Specificity	99	96	98
	PVN	90	84	81
N		6846	4431	3748

EA, Non-Hispanic European American, AA, Non-Hispanic African American, MA, Mexican American

The mean difference between self-reported and measured BMI was greater in Mexican Americans (men -0.37, women -0.76 kg/m^2^) than in non-Hispanic EA (men -0.22, women -0.62 kg/m^2^). In a regression model with the difference between self-reported and measured BMI as the dependent variable and MA ethnicity as the exposure variable and controlling for demographic variables or demographic variables plus smoking and health status, MA ethnicity was a significant predictor of BMI difference (Table 7).

**Table 7:** Adjusted regression coefficients (SE) of Mexican American ethnicity (yes/no) as a predictor of difference of measured and self-reported BMI by gender in NHANES III

Variable	N	Demographic-Adjusted*	95% CI	Demographic-and health-adjusted+	95% CI
Men	7552	1.28**	0.87–1.69	1.20**	0.81–1.59
					
Women	8142	2.35**	1.68–3.03	2.19**	1.51–2.87

CI, confidence interval

*adjusted for age (y), education < 12 years (yes/no), marital status (married/single), region (South vs other), metropolitan residence (yes/no).

+Adjusted for the above plus smoking status (smoker/nonsmoker) and poor self-reported health (yes/no)

** p < 0.01

An examination of mean differences between measured height and self-reported height indicated that MA men reported themselves to be 0.59 cm, EA men 1.47 cm and AA men 1.16 cm taller than measured. Over-reporting was greater at age 60+y than <60y, e.g. MA 60+ 2.00 cm, 20–59y 0.44 cm. MA men reported themselves to be 0.51 kg, EA men 0.26 kg and AA men 1.09 heavier than measured. Thus underestimates of overweight prevalence in MA men were due to over-reporting of height rather than underreporting of weight.

In women, MA reported themselves to be 0.51 cm, EA 0.26 cm and AA 1.09 cm taller than measured. Over-reporting was greater at age (years) 60+ than <60; e.g. MA 60+ 3.02 cm, 20–59 0.92 cm. MA women reported themselves to be 1.07 kg, EA women 1.21 kg, and AA women 1.58 kg lighter than measured. Under-reporting of weight was greater at age 20–59 than at 60+; e.g. MA 20–59, 1.18 kg; 60+, 0.28 kg. So underestimates of overweight prevalence in each group of women was due primarily to underreporting of weight at younger ages and both to over-reporting height and under-reporting weight at older ages.

## Discussion

Overweight/obesity is one of the leading preventable causes of death in the United States and most industrialized countries [1-7,23-26,32-34]. After release of national data from NHANES III (1988–1994) showed striking increases in US obesity prevalence among children and adults compared to earlier surveys, it was widely recognized that an "epidemic" was occurring [23-26]. Therefore monitoring of the prevalence of obesity by interview surveys, such as the National Health Interview Survey (NHIS) and the Behavioral Risk Factor Surveillance System (BRFSS), which rely on self-reported data, have assumed even greater importance than previously [34]. Yet, all current guidelines for the diagnosis and management of obesity are based on measured height and weight.

Over-sampling of the MA population and availability of measured as well as self-reported height and weight data in NHANES III made it possible to evaluate the ethnic variation in validity of self-reported data. NHANES III data show that using self-reported data produces consistent underestimates of the prevalence of overweight and obesity (BMI >= 25 kg/m^2^) in MA by up to 21 percentage points and of obesity (BMI >= 30 kg/m^2^) by up to 14 percentage points (Table 1). Criteria for deciding whether SR data are to be considered valid will depend on the application and on a number of factors including statistical test characteristics and expert judgement as discussed at length elsewhere [35]. However, these data suggest that self-reported data for MA are not sufficiently valid for use in producing such estimates and are suspect for etiologic research. Further research is needed determine the applicability of these findings to data from telephone interviews or self-completed questionnaires.

At a mechanistic level, the important underestimate of BMI and hence overweight and obesity prevalence in NHANES in women was due primarily to under-reporting of weight at younger ages and both to over-reporting height and under-reporting weight at older ages. Possible explanations for under-reporting of weight in women have been discussed [8-19]. In MA women and men, these may include recall bias and lack of information because of no recent measurements at home or by health care providers. This is important for both weight and height, since stature declines with age in older persons by up to 2 cm per decade after age 30 [8]. Lack of access to scales and health care and/or lack of utilization of either may be due to barriers such as cost, transportation, language or cultural factors. Poorer validity of self-reported hypertension has been noted in MA, perhaps for similar reasons [20]. Further, if obesity and short stature are seen as socially undesirable, a conscious or unconscious tendency to under-report body weight and over-report height may occur even if true weight and height are known. On the other hand, MA women have been reported to be less likely to perceive themselves as overweight and more satisfied with body size than non-Hispanic EA women in some, but not all studies [36-39]. Specifically in NHANES III, participants were asked, "Do you consider yourself now to be overweight, underweight, or about the right weight?" Hispanic women and men were more likely to under-assess their overweight status than non-Hispanic EA; e.g. 31% of overweight Hispanic women said they were "about the right weight" compared to 14% of non-Hispanic EA [37].

### Comparisons with previous reports

NHANES III is one of the largest studies to provide population-based data on the validity of prevalence estimates based on self report and one of the first to provide such data for Mexican Americans. Two previous reports were based on a nationally representative sample of US adults. One used NHANES III data to assess the effect of age on validity of self-reported data [8]. Under-reporting of height and consequently BMI bias was higher above age 60 than below. However, data from all ethnic groups were combined. The present report demonstrates important, consistent ethnic differences between MA and other groups. Patterns of reporting error in weight by gender and age were similar in NHANES II and III [8,9]. A non-significant effect of race ("white"/"black") on reporting bias for both weight and height was observed in NHANES II. In European and Australian adults, height was over-reported and weight under-reported, producing under-estimates of BMI and obesity prevalence [15-19]. An exception was the under-reporting of both weight and height in Scotland leading to BMI and prevalence estimates slightly higher than from measurement [10]. Brazilian adults reported height and weight with small errors that differed by gender [11]. In studies of American adolescents, height was over-reported and weight and consequently BMI and obesity prevalence under-reported [14]. The latter two studies did not describe ethnic variation. One report indicates that correction equations do not eliminate systematic error in self-reported BMI [40]. Comparisons of self-reported and measured BMI in etiologic research was beyond the scope of the present paper [41].

Among these studies, self-reported height and weight were obtained by in-person interview in several [8,9,11,13], by telephone interview [18], and by self-completed questionnaire others [10,14-17]. No study used more than one of these methods for self-reported data. Quantitative comparisons of error magnitude of self-reported data among studies are not possible due to varying designs, analytic methods, and reporting. A large British survey that used a self-completed questionnaire found mean differences between self-reported and measured BMI that ranged for -0.55 in women 35–49 years to -1.13 in men 60+ years, somewhat larger than in NHANES. An Australian telephone survey found differences in prevalence of overweight or obesity (percentage points) of -23 in men and -15 in women, much larger than those see in EA in NHANES. Variation in differences by ethnicity was not examined in other studies.

### Limitations and strengths

Several unavoidable limitations of the present study include possible bias arising from survey non-response and from missing values for some variables. Several special studies of NHANES III data have indicated little bias due to non-response [42]. The 2 to 4 week time lapse between ascertainment of self-reported data and measured data is unlikely to affect the difference in this adult population [8]. Compared to interview surveys such as NHIS or BRFSS, error in self-reported height and weight might be underestimated in NHANES III if subjects were aware before the interview that they would be weighed and measured during the subsequent examination and hence might be less likely to misstate height and weight. At the time of the home interview when participants reported their current height and weight, participants had not been invited to the NHANES III examination or told that the examination would include height and weight measurements. However, because of pre-survey media publicity or explicit questions asked of interviewers, some participants might have known or assumed they would later be measured and weighed. It is not possible to determine what effect, if any, this may have had on height and weight reporting.

Confounding by variables not controlled for cannot be excluded. However, given the uncertainty about the existence or nature of the association, it is unclear which other variables should be controlled for as confounders. The number of tests was restricted to those of weighted regression models. The representativeness of the sample and the use of sample weights provide generalizability of the results to United States non-institutionalized population of the same ages, but not necessarily to Mexico or other nations or smaller US ethnic groups such as Cubans.

In order to monitor overweight prevalence in Cuban and Puerto Rican Americans and American Indians using interview survey data, replication of the current findings in these groups is needed. Participants might be asked when their weight and their height were last measured and by whom. The validity of self-report data obtained by in-person interview, telephone interview, and self-completed questionnaire should be compared to establish generalizablity of validity estimates from the former to the latter two. Trends in validity over time should also be sought in MA using more recent surveys and related to possible correlates.

## Conclusion

Self reported height, weight and BMI in Mexican Americans may not be of sufficient validity for use by public health agencies and they underestimate obesity prevalence in women of all ethnic groups.

## List of Abbreviations

AA, African American

BMI, body mass index

EA, European American

MA, Mexican American

NHANES, National Health and Nutrtion Examination Survey

## Supplementary Material

Additional File 1 Appendix Tables 1 and 2 Click here for file
