# Supplementary material for: Ethnic variation in validity of classification of overweight and obesity using self-reported weight and height in American women and men: the Third National Health and Nutrition Examination Survey
Source: Nutr J. 2005 Oct 6;4:27. doi: 10.1186/1475-2891-4-27 (PMC1262765; doi:10.1186/1475-2891-4-27)
Supplement: Additional File 1 — Appendix Tables 1 and 2 [file 1475-2891-4-27-S1.doc]

SUPPLEMENTARY INFORMATION

APPENDIX

Table A1. Sensitivity of overweight or obesity from body mass index >= 25 kg/m^2^ based on self-reported height and weight by gender, age, smoking and ethnicity: NHANES III.(weighted)

|  |  | Ethnicity | | |  |
| --- | --- | --- | --- | --- | --- |
|  | Current smoker | EA | AA | MA | N |
| Men |  |  |  |  |  |
| 20-59y | Yes | 92 | 93 | 82 | 1850 |
|  | No | 93 | 92 | 83 | 3119 |
| 60+y | Yes | 84 | 91 | 79 | 496 |
|  | No | 87 | 88 | 83 | 2182 |
| Women |  |  |  |  |  |
| 20-59y | Yes | 84 | 87 | 70 | 1491 |
|  | No | 89 | 90 | 72 | 4043 |
| 60+y | Yes | 83 | 85 | 82 | 328 |
|  | No | 79 | 82 | 66 | 2511 |
|  |  |  |  |  |  |

EA, Non-Hispanic European American, AA, Non-Hispanic African American, MA, Mexican American

*>=24 times/year

WEIGHTED

Table A2. Percentage of persons with self-reported body mass index (BMI) < 25 kg/m^2^ (not overweight) who were overweight or obese (BMI >= 25 kg/m^2^) by measured BMI by gender, age, smoking and ethnicity: NHANES III.(weighted)

|  |  | Ethnicity | | |  |
| --- | --- | --- | --- | --- | --- |
|  | Current smoker | EA | AA | MA | N |
| Men |  |  |  |  |  |
| 20-59y | Yes | 8.8 | 6.7 | 26.6 | 1850 |
|  | No | 10.9 | 15.2 | 26.3 | 3119 |
| 60+y | Yes | 17.2 | 7.7 | 22.5 | 496 |
|  | No | 24.7 | 21.0 | 33.3 | 2182 |
| Women |  |  |  |  |  |
| 20-59y | Yes | 10.0 | 15.7 | 35.7 | 1491 |
|  | No | 8.2 | 18.5 | 36.4 | 4043 |
| 60+y | Yes | 12.1 | 26.6 | 21.7 | 328 |
|  | No | 24.0 | 39.5 | 51.5 | 2511 |
|  |  |  |  |  |  |

EA, Non-Hispanic European American, AA, Non-Hispanic African American, MA, Mexican American

*>=24 times/year
